# Pregnancy Rate of Intrauterine Insemination (IUI) in an In Vitro Fertilization (IVF) Unit in King Abdulaziz Medical City, Riyadh

**DOI:** 10.7759/cureus.79102

**Published:** 2025-02-16

**Authors:** Zeyad Abualiat, Dalal Alanazi, Mashael Alanazi, Maha Alwan, Reem Yaqoub, Alhanoof Alhomrani, Nouf Alsomali

**Affiliations:** 1 Obstetrics and Gynaecology, King Abdulaziz Medical City, Riyadh, SAU; 2 Obstetrics and Gynaecology, Dr. Sulaiman Al-Habib Medical Group, Riyadh, SAU; 3 College of Medicine, King Saud Bin Abdulaziz University for Health Sciences, Riyadh, SAU

**Keywords:** daterminants, intrauterine insemination, in vitro fertilization, pregnancy rate, saudi arabia

## Abstract

Background: Among the numerous studies investigating the success rate of intrauterine insemination (IUI), there is a dearth of reliable and applicable data. This retrospective cohort study targeting women in infertile couples aims to provide insight into the local success rate of IUI and its determinants.

Objectives: To determine the success (pregnancy) rate of IUI and its determinants among infertile couples.

Material and methods: A retrospective cohort study was conducted on women in infertile couples at the King Abdulaziz Medical City (KAMC) in-vitro fertilization (IVF) unit in Riyadh, Saudi Arabia. A total of 238 patients were selected utilizing a non-probability consecutive sampling technique. The success of IUI was determined by the percentage of couples who attained a clinical pregnancy after undergoing the procedure. Data was collected and analyzed using the Statistical Package for Social Sciences (SPSS).

Results: The study included 238 women. Their age ranged between 21 and 49 years with an arithmetic mean±SD of 31.6±5.1 years. Regarding causes of infertility, female factors were responsible for 33.2% of cases whereas, in 59.6% of cases, the cause was described as unexplained. The success rate of IUI was 13.4%. Women’s gravidity and parity were the only factors significantly associated with the success of IUI as the highest success rate was reported among women with gravidity of 2 (27.6%) and parity of 2 (36%). In contrast, the lowest rates were reported among those of gravidity of 1 (5.9%) and parity of ≥3 (5.9%) with p-values of 0.010 and 0.005, respectively.

Conclusion: When compared to other fertility treatments, the success rate of IUI is relatively low; however, it is comparable to previously reported IUI success rates in other centers in developed countries. It is recommended that a large multi-center study with a bigger sample size be conducted to determine the accuracy on a larger scale. The findings from this study carry valuable implications for guiding the selection of suitable fertility treatments.

## Introduction

One of the varied treatments provided to infertile couples seeking to conceive is Intrauterine insemination (IUI). This is a widely used fertility treatment that involves directly inserting sperm into the uterus to facilitate fertilization. The success of IUI is dependent on the etiology of infertility [1]. This procedure is considered when the conceiving couple faces mild male factor infertility, cervical factor infertility, or unexplained infertility. It is often performed at in vitro fertilization (IVF) facilities as a less invasive and more cost-effective alternative to IVF [1,2].

The success of IUI is determined by the pregnancy rate, which refers to the percentage of couples who achieve a clinical pregnancy following the procedure [2]. Multiple factors play a role in the conception rate after IUI, such as the quality of sperm preparation, ovarian stimulation protocols, timing of insemination, and the etiology of infertility [3].

According to a recent study that focused on predictive factors for intrauterine insemination outcomes, advancing maternal and paternal age has a negative impact on conception rates. Elevated maternal BMI increases medication doses without affecting pregnancy outcomes; however, paternal obesity is a contributing factor to infertility. Notably, the majority of successful pregnancies occur within the first four cycles of IUI [3].

Globally, the overall pregnancy rate following IUI ranges significantly, from as low as 2.7% to as high as 66%, underscoring the variability in outcomes across different regions and patient populations. These findings emphasize the multifaceted nature of factors influencing IUI success rates and highlight the importance of tailored approaches in managing infertility treatments [1,2,4].

According to the local data that was made in 2020 in which they examined the cost-effectiveness of assisted reproductive technologies in Saudi Arabia, the live birth rate per initiated IUI cycle was 7.9% [3]. Another study conducted in the southern region of Saudi Arabia demonstrated that the augmentation of the luteal phase with vaginal progesterone combined with ovulation induction via recombinant follicle-stimulating hormone has improved the success rate of IUI cycles [4]. Other locally conducted studies showed a pregnancy success rate of 16% in Tabuk and 7.9% in Qassim [5,6].

After an extensive literature review, it has become evident that while studies exist on the success rates of IUI, there is a notable lack of local research providing reliable and applicable data. This research aims to address this gap by specifically investigating the success rate of IUI and its determinants among infertile couples in our local context. By elucidating the factors influencing the success of IUI, this research aims to assist in tailoring treatment options and enhancing outcomes for couples striving to achieve conception. The results of this study will contribute to the existing body of knowledge on fertility treatments, aiding healthcare professionals in delivering personalized and evidence-based care to infertile couples and in effectively counseling patients to manage their expectations.

## Materials and methods

A retrospective cohort study was conducted at the King Abdulaziz Medical City in Riyadh (KAMC-R), Saudi Arabia where the IVF unit served as the focal point for the investigation. The unit specializes in addressing various types of infertility, including primary and secondary infertility caused by male-factor infertility, female-factor infertility, or unexplained infertility. On average, the unit receives approximately 600 new patient visits per year, with an admission rate ranging from 6 to 12 per year [7].

The study encompassed all women in couples with different causes of infertility (male factor, female factor, or unexplained) who sought treatment at the IVF unit in KAMC-R, between January 2019 and September 2023 and underwent IUI. The inclusion criteria comprised couples meeting these conditions, while the exclusion criteria comprised couples who did not fall within the specified date range. Controlled ovarian stimulation using gonadotropins (Gonal F, Menogon, Merional, and others) was performed prior to IUI.

Data was retrieved from the Best Care system in National Guard Health Affairs (NGHA)-Riyadh using a non-probability consecutive sampling technique. The collected data, inclusive of patient demographics, body mass index (BMI), insemination dates, previous inseminations, comorbidities, and insemination outcomes (failed pregnancy, intrauterine pregnancy, ectopic pregnancy, or abortion), were then securely stored at the IVF unit database.

The data was then analyzed using the Statistical Package for Social Science (SPSS) software (IBM Corp. Released 2021. IBM SPSS Statistics for Windows, Version 28.0. Armonk, NY: IBM Corp) for statistical analysis. Frequency and percentage were utilized for describing categorical data, while the arithmetic mean and standard deviation (SD) were employed for continuous numerical data. The outcome variable of interest was the number of successful births resulting from IUI. Statistical analyses involved the use of the Chi-square test or Fisher’s exact test for comparing categorical variables and the Student's t-test for comparing continuous variables, with statistical significance set at a P value less than 0.05.

Ethical approval for the study was granted by the King Abdullah International Medical Research Center (KAIMRC) in Riyadh, Saudi Arabia, with the Institutional Review Board (IRB) approval number IRB/2765/23 on November 5th, 2023.

## Results

The study included 238 women. The demographic, obstetric, and baseline characteristics of the study population are summarized in Table 1. The age ranged between 21 and 49 years with an arithmetic mean±SD of 31.6±5.1 years. The majority of them (92.9%) were below 40 years old. Obesity was reported among 39.5% of them. Their gravidity and parity exceeded 3 in 16.8% and 7.1% of cases, respectively. Almost half of the cases (49.2%) were of secondary infertility. Regarding the cause of infertility, female factors were responsible for 33.2% of cases, whereas in 59.6% of cases, the cause was described as unexplained. The duration of infertility ranged between one to three years in 42.5% of cases. About 13.4% of them had two or more previous IUI cycles. Endometrial thickness ranged between 3.0-18.6 mm with an arithmetic mean±SD of 9.7±2.5 mm. Co-morbidity was reported among almost half (49.1%) of cases. Sperm concentration ranged between 10 and 212 with an arithmetic mean±SD of 51.4±31.8 million/milliliter, while sperm motility ranged between 7 and 99% with an arithmetic mean±SD of 62.4±18%.

**Table 1:** Demographic, obstetric and baseline characteristics of the women who underwent intrauterine insemination at King Abdulaziz Medical City between January 2019 and September 2023. SD: Standard deviation IUI: Intrauterine Insemination

Characteristics	Frequency	Percentage
Age (years) “n=238”		
<40	221	92.90%
≥40	17	7.10%
Range	21 - 49	
Mean ±SD	31.6 ± 5.1	
Body mass index (Kg/m^2^)		
Underweight	5	2.10%
Normal	63	26.50%
Overweight	76	31.90%
Obese	94	39.50%
Gravidity		
0	118	49.60%
1	51	21.40%
2	29	12.20%
≥3	40	16.80%
Parity		
0	150	63.10%
1	46	19.30%
2	25	10.50%
≥3	17	7.10%
Type of infertility		
Primary	121	50.80%
Secondary	117	49.20%
Cause of infertility		
Male factor	14	5.90%
Female factor	79	33.20%
Both	3	1.30%
Unexplained	142	59.60%
Duration of infertility (years)		
≤1	20	8.40%
>1-3	101	42.50%
>3-5	66	27.70%
>5	51	21.40%
Endometrial thickness (mm) “n=237”		
Range	3.0 - 18.6	
Mean ±SD	9.7 ± 2.5	
Co-morbidity		
None	121	50.90%
Gynecological	41	17.20%
Non-gynecological	70	29.40%
Both	6	2.50%
Number of previous IUI cycles		
0	154	64.80%
1	52	21.80%
≥2	32	13.40%
Sperm concentration (million/milliliter)		
Range	10 - 212	
Mean ±SD	51.4 ± 31.8	
Sperm motility (%)		
Range	7 - 99	
Mean ±SD	62.4 ± 18.0	

 Regarding the type of induction, the majority of them (89.9%) had Gonal F type as seen in Table 2.

**Table 2:** Distribution of the women who underwent intrauterine insemination at King Abdulaziz Medical City between January 2019 and September 2023 according to type of induction.

Type of induction	Percentage %
Gonal F	89.9
Menogon	5.4
Merional	4.6
Others	1.2

The success rate of intrauterine insemination, determined by the occurrence of pregnancy, was 13.4% (Table 3). Women’s gravidity and parity were the only factors significantly associated with the success of IUI with the highest success rate reported among women with gravidity of 2 (27.6%) and parity of 2 (36%). On the other hand, the lowest rates were reported among those of gravidity of 1 (5.9%) and parity of ≥3 (5.9%) with p-values of 0.010 and 0.005, respectively (Table 4, 5).

**Table 3:** Outcome of intrauterine insemination at King Abdulaziz Medical City between January 2019 and September 2023, determined by occurrence of pregnancy.

Outcome	Number of patients	Percentage
Success	32	13.4%
Failure	206	86.6%

**Table 4:** Factors associated with success of intrauterine insemination, King Abdulaziz Medical City (January 2019 and September 2023) **Chi-square test ˚Fischer Exact test *Independent two sample t-test IVF: In vitro fertilization

Parameters	Outcome of intrauterine insemination N%		Test statistic	p-value
	Failure N=206	Success N=32		
Age (years)				
<40 (n=221)	190 (86.0)	31 (14.0)	---	0.302˚
≥40 (n=17)	16 (94.1)	1 (5.9)		
Body mass index (Kg/m^2^)				
Underweight (n=5)	5 (100)	0 (0.0)	0.654 (df=3)	0.701**
Normal (n=63)	54 (85.7)	9 (14.3)		
Overweight (n=76)	64 (84.2)	12 (15.8)		
Obese (n=94)	83 (88.3)	11 (11.7)		
Gravidity				
0 (n=118)	106 (89.8)	12 (10.2)	11.396 (df=3)	0.010**
1 (n=51)	48 (94.1)	3 (5.9)		
2 (n=29)	21 (72.4)	8 (27.6)		
≥3 (n=40)	31 (77.5)	9 (22.5)		
Parity				
0 (n=150)	132 (88.0)	18 (12.0)	12.925 (df=3)	0.005**
1 (n=46)	42 (91.3)	4 (8.7)		
2 (n=25)	16 (64.0)	9 (36.0)		
≥3 (n=17)	16 (94.1)	1 (5.9)		
Type of infertility				
Primary (n=121)	109 (90.1)	12 (9.9)	2.633 (df=1)	0.105**
Secondary (n=117)	97 (82.9)	20 (17.1)		
Cause of infertility				
Male factor (n=14)	12 (85.7)	2 (14.3)	2.222 (df=3)	0.528**
Female factor (n=79)	66 (83.5)	13 (16.5)		
Both (n=3)	2 (66.7)	1 (33.3)		
Unexplained (n=142)	126 (88.7)	16 (11.3)		
Duration of infertility (years)				
≤1 (n=20)	16 (80.0)	4 (20.0)	1.491 (df=3)	0.684**
>1-3 (n=101)	86 (85.1)	15 (14.9)		
>3-5 (n=66)	59 (89.4)	7 (10.6)		
>5 (n=51)	45 (88.2)	6 (11.8)		
Endometrial thickness (mm) “n=237”				
<8 (n=54)	45 (83.3)	9 (16.7)	0.6 (df=1)	0.439*
≥8 (n=183)	160 (87.4)	23 (12.6)		
Co-morbidity				
None (n=121)	104 (86.0)	17 (14.0)	1.269 (df=3)	0.736**
Gynecological problems (n=41)	34 (82.9)	7 (17.1)		
Non-gynecological problems (n=70)	63 (90.0)	7 (10.0)		
Both (n=6)	5 (83.3)	1 (16.7)		
Number of IVF previous cycles				
0 (n=154)	132 (85.7)	22 (14.3)	0.281 (df=2)	0.869**
1 (n=52)	46 (88.5)	6 (11.5)		
≥2 (n=32)	28 (87.5)	4 (12.5)		
Type of induction				
Gonal F (n=213)	185 (86.9)	28 (13.1)	0.157 (df=1)	0.442˚
Human menopausal gonadotropine (n=25)	21 (84.0)	4 (16.0)		

**Table 5:** Factors associated with success of intrauterine insemination, King Abdulaziz Medical City (January 2019 and September 2023) *Independent two sample t-test

Mean±SD	Outcome of intrauterine insemination		Test statistic	p-value
	Failure	Success		
Endometrial thickness (mm)	9.7±2.5 (n=205)	10.1±2.6 (n=32)	0.944 (df=235)	0.346*
Sperm concentration(million/milliliter)	50.5±31.6 (n=203)	57.2±33.3 (n=30)	1.078 (df=231)	0.282*
Sperm motility (%)	61.9±18.4 (n=203)	65.8±15.4 (n=30)	1.109 (df=231)	0.269*

## Discussion

As there are inconsistencies among studies in the literature on the IUI success rate and its determinants, the present study tried to investigate this in a high-quality in-vitro fertilization (IVF) unit in Riyadh, Saudi Arabia.

This study revealed a success rate of 13.4% with IUI as determined by the occurrence of pregnancy as 13.4%. Comparable rates have been reported by others. A higher (18%) rate has been reported by Osaikhuwuomwan et al. in Nigeria [8]. In the United States, viable birth was observed in 9.4% of 2912 IUI cycles in 1117 women [9]. Comparison between these studies and the present one should be taken in the light of differences in the demographic characteristics of the participants in these studies. However, in all studies, the success rate of IUI is considered low. 

Our research revealed that a woman's history of pregnancies and live births, referred to as gravidity and parity, plays a significant role in the success of Intrauterine Insemination (IUI). Notably, women with previous pregnancies or live births had the highest success rates. This finding is consistent with the work of Geisler et al. which also identified previous parity as a critical factor for successful IUI outcomes [10]. These results indicate that a woman's past pregnancies can influence the effectiveness of IUI. This effect is likely related to factors such as reproductive health and the receptivity of the uterus.

Many studies showed that the success rate of IUI was observed more in younger women [8,9,11-15]. Although our study reported a higher success rate among women aged below 40 years compared to those aged 40 years or above (14% vs. 5.9%), this difference was not statistically significant. This could be partially attributed to the fact that only 7.1% of our population was aged 40 years or above. It has been recommended that, for women over 35, IUI as a treatment option needs careful consideration, and for those over 40, it is not recommended [16,17].

In disagreement with others, the present study did not observe a difference in the IUI success rate according to women’s body mass index [9,15]. Souter et al. observed that an increase in women’s BMI was associated with fewer follicles produced per given follicular stimulating hormone dose; however, there was no difference in number of IVF cycles required to conceive or the clinical pregnancy rate [18].

Many studies have shown that endometrial thickness is a significant determinant of successful IUI [19,20]. Furthermore, the highest rate of clinical pregnancy was considered with an endometrial thickness of 8-12 mm [21]. Weissman et al. observed that the pregnancy rates were higher with an endometrial thickness of 7-14 mm than with >14 mm while Zhao et al. observed that the clinical pregnancy rate was significantly higher with an endometrial thickness of >7 mm than with <7 mm [22,23]. Conclusively, a thin endometrium may reduce the pregnancy rate. In the current study, although endometrial thickness was higher in success than failure groups of IUI, this was not statistically significant. The same has been observed by Wang et al [11].

Some investigators reported that most pregnancies occur within the first four IUI or IVF cycles [24-26]. In the current survey, number of IUI cycles was not a predictive factor for successful IUI. It has been documented that the decision to continue IUI cycles should be taken in light of female age, cost of therapy, and goals of treatment [3].

The study has some important limitations that should be mentioned. First, it was conducted at a single health center, which could impact the ability to generalize its findings over other healthcare centers in Riyadh and Saudi Arabia. Second, having a limited number of women aged over 40 years might influence the impact of women’s age on the success of IUI. The retrospective nature of the study and the accuracy of medical records is considered a limitation of the study as it is subjected to bias. Due to a small number of significant factors, we could not perform multivariate logistic regression analysis to control for confounders. Despite these limitations, the study represents a good experience in one of the largest IVF centers in the capital of Saudi Arabia in the field of IUI, which might have significance in patient counseling concerning IUI treatment.

## Conclusions

The findings of this study underscore the consistent challenge of the relatively low success rates of IUI, a trend mirrored in comparable centers across developed countries. The pivotal role of women's gravidity/parity in influencing IUI outcomes highlights the need for a more expansive multi-center study, emphasizing the inclusion of a sufficient number of women aged over 40 years. This recommendation aims to deepen our understanding of factors impacting IUI success, ultimately enhancing patient care and counseling in the realm of assisted reproductive technologies.

The insights gained from this paper have significant implications when it comes to recommending appropriate fertility treatments. By recognizing the influence of gravidity/parity on IUI success, healthcare providers can tailor treatment strategies more effectively. A broader multi-center study, especially encompassing older women, can refine treatment protocols and decision-making processes, leading to more personalized and successful outcomes in fertility interventions. This research paves the way for improved patient management and underscores the importance of comprehensive studies in shaping the landscape of fertility treatment recommendations.
